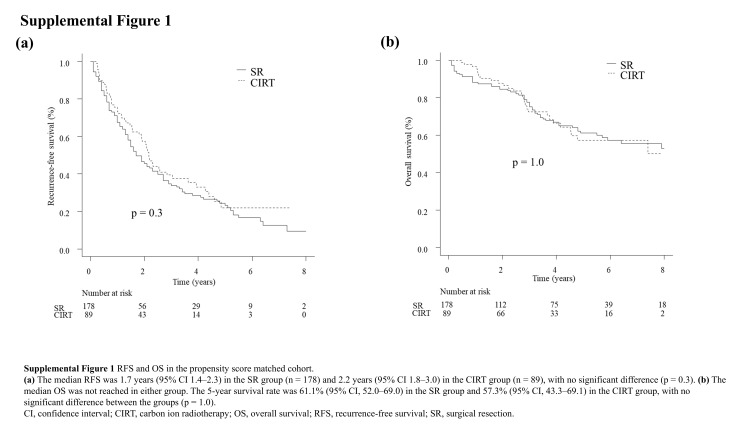# Carbon-ion radiotherapy achieves outcomes equivalent to surgical resection for hepatocellular carcinoma

**DOI:** 10.1097/HC9.0000000000000801

**Published:** 2025-08-29

**Authors:** Takeshi Hatanaka, Kei Shibuya, Satoru Kakizaki, Atsushi Hiraoka, Toshifumi Tada, Kazuya Kariyama, Ei Itobayashi, Kunihiko Tsuji, Toru Ishikawa, Hidenori Toyoda, Yuichi Koshiyama, Atsushi Naganuma, Yuhei Miyasaka, Yuki Kanayama, Kazunari Tanaka, Fujimasa Tada, Hideko Ohama, Kazuhiro Nouso, Shinichiro Nakamura, Takashi Kumada, Tatsuya Ohno

**Affiliations:** 1 Department of Gastroenterology, Gunma Saiseikai Maebashi Hospital, Maebashi, Japan; 2 Department of Radiation Oncology, Gunma University Graduate School of Medicine, Maebashi, Japan; 3 Department of Clinical Research, NHO Takasaki General Medical Center, Takasaki, Japan; 4 Department of Gastroenterology and Hepatology, Gunma University Graduate School of Medicine, Maebashi, Japan; 5 Gastroenterology Center, Ehime Prefectural Central Hospital, Matsuyama, Japan; 6 Department of Internal Medicine, Division of Gastroenterology, Kobe University Graduate School of Medicine, Kobe, Japan; 7 Department of Gastroenterology, Okayama City Hospital, Okayama, Japan; 8 Department of Gastroenterology, Asahi General Hospital, Asahi, Japan; 9 Center of Gastroenterology, Teine Keijinkai Hospital, Sapporo, Japan; 10 Department of Gastroenterology, Saiseikai Niigata Hospital, Niigata, Japan; 11 Department of Gastroenterology and Hepatology, Ogaki Municipal Hospital, Ogaki, Japan; 12 Department of Gastroenterology, NHO Takasaki General Medical Center, Takasaki, Japan; 13 Department of Internal Medicine, Japanese Red Cross Society Himeji Hospital, Himeji, Japan; 14 Gifu Kyoritsu University, Ogaki, Japan

**Keywords:** charged particle therapy, curative treatment, early stage, liver resection, solitary

## Abstract

**Aim:**

This study aimed to compare the clinical outcomes of carbon-ion radiotherapy (CIRT) to those of surgical resection (SR) in patients with HCC.

**Methods:**

This retrospective study included 116 and 947 patients initially receiving CIRT and SR in Japanese institutions from September 2010 and June 2022. We used inverse probability of treatment weighting (IPTW) analysis to correct for imbalances in baseline patient characteristics between the 2 groups.

**Results:**

The median observation period was 3.3 years (IQR: 1.4–5.8) in the SR group and 2.8 years (IQR: 1.6–4.5) in the CIRT group (*p*=0.2). Before IPTW analysis, the median recurrence-free survival (RFS) was 2.3 years in the SR group and 2.2 years in the CIRT group, with no statistical significance (*p*=0.3). After IPTW analysis, the median RFS was 2.5 years in the SR group and 2.3 years in the CIRT group, which remained statistically nonsignificant (*p*=0.9). The median overall survival (OS) was not reached in the SR group, while it was 7.4 years in the CIRT group. The SR group demonstrated better survival compared to the CIRT group (*p*=0.02). In the IPTW cohort, the median OS was not reached in the SR group, while it remained 7.4 years in the CIRT group, showing no significant difference (*p*=0.4). Multivariate analyses showed that treatment choice (SR vs. CIRT) was not identified as a predictive factor for both RFS and OS.

**Conclusions:**

CIRT showed no statistically significant differences in RFS or OS compared with SR, suggesting its potential as a curative treatment option for early-stage HCC.

## Graphical abstract

**Figure:**
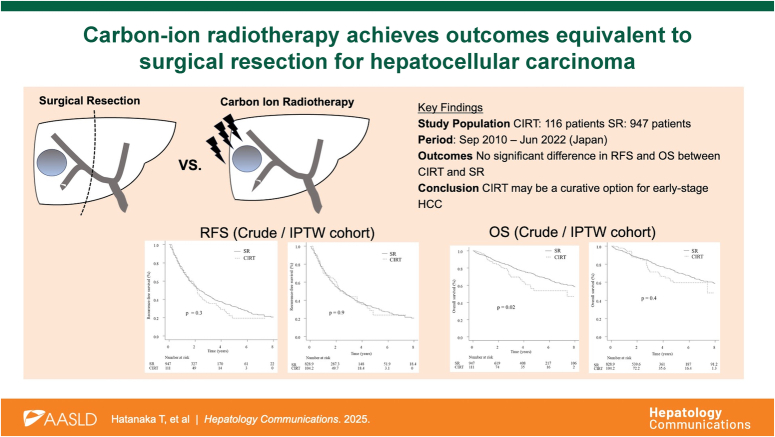

## INTRODUCTION

Approximately 0.86 million individuals are newly diagnosed with liver cancer each year, making it the second leading cause of cancer-related mortality worldwide.^1^ HCC, the most common form of liver cancer, typically arises in the context of chronic liver diseases, including hepatitis B or C virus infection, habitual alcohol consumption, or steatohepatitis. Liver resection, liver transplantation, and ablation therapy are well-established curative options for early-stage HCC. According to the American Association for the Study of Liver Diseases (AASLD) and the European Association for the Study of the Liver practice guidelines,^2^,^3^ liver resection is recommended for solitary or localized HCC in patients with cirrhosis with well-preserved liver function and no significant portal hypertension, as well as in patients without cirrhosis. However, liver resection is not always feasible due to factors such as the tumor’s anatomical location, the planned extent of hepatectomy, the expected residual liver volume, and the presence of comorbidities.

Radiation therapy, including stereotactic body radiotherapy (SBRT)^4^,^5^ and charged particle therapy,^6^ has emerged as a promising treatment modality for HCC. Carbon-ion radiotherapy (CIRT), in particular, offers superior dose distribution and high biological effectiveness compared to x-ray–based therapies. Several prospective studies^7^,^8^,^9^ have demonstrated that CIRT achieves good local tumor control with an acceptable safety profile. Nevertheless, whether CIRT can be considered a standard treatment for HCC remains unclear, as comparative studies assessing its efficacy against established treatments, particularly surgical resection (SR), are limited. This study aims to evaluate the clinical outcomes of CIRT in comparison with SR.

## METHODS

### Patients

**FIGURE 1:**
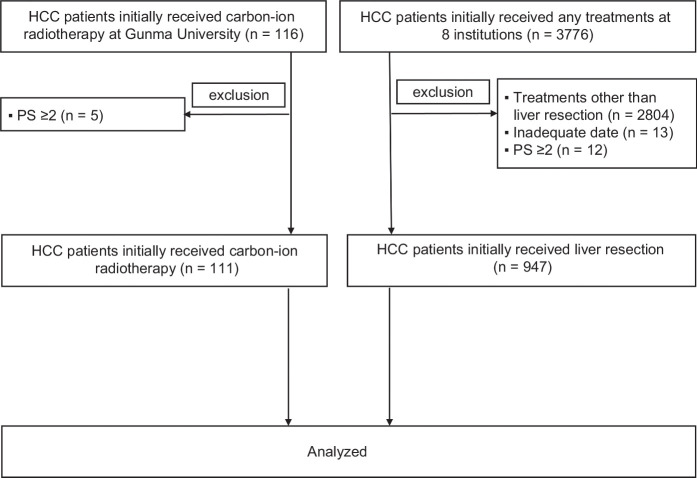
Patient selection flowchart. Abbreviation: PS, performance status.

This retrospective multicenter study included patients based on the following criteria: (1) liver function classified as Child-Pugh class A or B, (2) performance status (PS) of 0 or 1, and (3) initial treatment with either SR or CIRT. Patients with incomplete or inadequate data were excluded. Between September 2010 and June 2022, a total of 116 patients with HCC were initially treated with CIRT at Gunma University. Among these, 5 patients were excluded due to a PS of ≥2, leaving 111 patients for inclusion in the CIRT group. During the same period, 3776 treatment-naive patients with HCC underwent various therapies across 8 Japanese institutions. Among these, we excluded patients who received treatments other than SR (n=2804), those with inadequate data (n=13), and those with a PS of ≥2 (n=12). Consequently, 947 patients were included in the SR group. No patients received systemic therapy before CIRT or SR, nor were they scheduled to receive systemic therapy following these treatments until disease recurrence. In addition, no patients received additional local therapies as part of the initial treatment strategy. The patient selection process is detailed in Figure 1. This retrospective study was approved by the Institutional Ethics Committee of Gunma Saiseikai Maebashi Hospital (IRB No. 2023-16) in accordance with the Declarations of Helsinki and Istanbul. Informed consent was obtained using an opt-out approach, as approved by the ethics committee.

HCC was diagnosed based on pathological findings and/or typical radiological imaging in accordance with the AASLD guidelines.^2^ Diagnostic radiological imaging criteria were conducted based on arterial-phase enhancement and washout during the portal venous or delayed phases on dynamic CT or MRI. Treatment decisions were generally made as follows: PS, liver function, and tumor spread were evaluated through physical examinations, laboratory data, and radiological imaging. Subsequently, treatment selection and implementation were determined through discussions with multidisciplinary teams at each local hospital.

### CIRT treatments

The CIRT treatment method was described elsewhere.^9^,^10^ Specifically, for treatment planning, a fiducial marker was implanted near the tumor under image guidance to identify its location. When suitable landmarks such as lipiodol deposition, embolic coils, or surgical clips were present, marker placement was unnecessary. Respiratory-gated CT was used for simulation and planning, with patients immobilized using cushions and 3-mm thermoplastic shells.

The gross tumor volume was delineated using respiratory-gated CT fused with contrast-enhanced CT or MRI. A 5-mm clinical target volume margin was applied but adjusted to avoid critical structures such as the digestive tract and portal vein branches. The planning target volume margin included the respiratory motion range and a 3-mm setup margin.

CIRT doses were expressed in Gy (relative biological effectiveness), calculated by multiplying the physical dose by the relative biological effectiveness of carbon-ion beams. Carbon-ion beams were delivered using a synchrotron at Gunma University Heavy-ion Medical Center, with energies of 290, 380, or 400 MeV/μ, selected based on tumor depth. The prescribed total dose was 52.8 or 60 Gy (relative biological effectiveness), delivered in 4 fractions for tumors separated from organs at risk (alimentary tract and porta hepatis). For tumors located <1 cm from the alimentary tract or extensively adjacent to the porta hepatis, 60 Gy was administered in 12 fractions.

Before each treatment, 2-directional x-rays were taken to verify patient alignment with the treatment plan. Any deviation ≥3 mm between the skeletal structure and the fiducial marker prompted corrections. Beams were delivered during the expiratory phase using a respiratory gating system (AZ-733 V; Anzai Medical).

The eligibility for CIRT was determined for all cases through a multidisciplinary board, including experienced hepatobiliary surgeons, hepatologists, interventional radiologists, and radiation oncologists at Gunma University.

### Statistical analyses

Numerical variables were expressed as medians with IQR and compared using the Mann-Whitney *U* test. Categorical variables were presented as numbers with percentages and compared using either the chi-squared test or the Fisher exact test, as appropriate. Recurrence-free survival (RFS) was defined as the period from the initiation of treatment to the occurrence of recurrence or death, whichever came first. Overall survival (OS) was defined as the interval between the initiation of treatment and death from any cause. To correct for imbalances in baseline patient characteristics between the 2 groups, we adopted inverse probability of treatment weighting (IPTW). Propensity scores for the initial treatment were calculated using a logistic regression model that included the following covariates: age, sex, PS, modified albumin-bilirubin (mALBI) grade, ALBI score, number of tumors (solitary vs. multiple nodules), etiology of chronic liver disease (viral vs. nonviral), tumor size, macroscopic vascular invasion (MVI), serum α-fetoprotein (AFP) ≥100 ng/mL, and serum des-gamma-carboxy prothrombin (DCP) ≥100 mAU/mL. Propensity scores in the bottom and top one percentiles were trimmed to avoid extreme weights. Inverse probability weights were then calculated for each patient. IPTW-adjusted Kaplan-Meier curves and weighted log-rank tests were applied to the weighted cohort. IPTW-adjusted Cox proportional hazard regression was used to estimate HRs. As a sensitivity analysis to assess the robustness of the IPTW results, we also performed 2:1 propensity score matching (PSM) using nearest-neighbor matching with a caliper of 0.2. A multiple imputation method was used to address the missing data. A Cox regression model was used to explore the predictive factors for RFS and OS. Univariate analyses were performed using the following covariates: age, sex, PS, etiology of chronic liver disease (viral vs. nonviral), mALBI grade, number of tumors (solitary vs. multiple nodules), tumor size, MVI, serum AFP ≥100 ng/mL, serum DCP ≥100 mAU/mL, and treatment modality (SR vs. CIRT). Variables with a *p*-value <0.10 in the univariate analysis were included in the multivariate analyses for RFS and OS. All statistical analyses were conducted using EZR Ver. 1.67 (Saitama Medical Center, Jichi Medical University).^11^

## RESULTS

### Patient characteristics

The median age was 71.0 years old (IQR: 65.0–77.0) and 74 years old (IQR: 67.0–82.0) in the SR and CIRT group, respectively. A total of 748 (79.0%) patients in the SR group and 81 (73.0%) patients in the CIRT group were male. PS was 0 in 890 patients (94.0%) and 1 in 57 patients (6.0%) in the SR group, whereas it was 0 in 71 patients (64.0%) and 1 in 40 patients (36.0%) in the CIRT group. The SR group demonstrated a better PS distribution than the CIRT group (*p*<0.001). Regarding chronic liver disease etiology in the SR group, 384 patients (40.6%) had HCV, 147 (15.5%) had HBV, 2 (0.2%) had both HCV and HBV, 97 (10.3%) had significant alcohol consumption, and 316 (33.4%) had other causes. In the CIRT group, 51 patients (45.9%) had HCV, 10 (9.0%) had HBV, 19 (17.1%) had significant alcohol consumption, and 31 (27.9%) had other causes. Approximately 90% of patients in both groups had Child-Pugh class A liver function. The median ALBI score was -2.78 (IQR: −3.04 to −2.50) in the SR group and −2.60 (IQR: −2.82 to −2.25) in the CIRT group, indicating significantly better liver function in the SR group (*p*<0.001). Consequently, the mALBI grade distribution in the SR group was grade 1 in 621 patients (65.6%), grade 2a in 194 (20.5%), grade 2b in 123 (13.0%), and grade 3 in 9 (1.0%). In the CIRT group, mALBI grades were grade 1 in 56 patients (50.5%), grade 2a in 25 (22.5%), and grade 2b in 30 (27.0%), showing superior liver function in the SR group (*p*=0.001). The number of patients with a solitary nodule was 729 (77.0%) in the SR group and 104 (93.7%) in the CIRT group. Solitary nodules were more frequent in the CIRT group than in the SR group (*p*<0.001). The median maximum tumor size was 3.3 cm (IQR 2.2–5.3) in the SR group and 3.7 cm (IQR 2.6–5.6) in the CIRT group. The number of patients with tumors larger than 4 cm in diameter was 369 (39.0%) in the SR group and 54 (48.6%) in the CIRT group. The baseline characteristics of patients in both groups are presented in Table 1.

**TABLE 1:** Patient characteristics in the SR and CIRT groups

Factor	SR group (n=947)	CIRT group (n=111)	*p*
Age (y)			
Median (IQR)	71.0 [65.0, 77.0]	74.0 [67.0, 82.0]	<0.001
Gender, n (%)			
Male	748 (79.0)	81 (73.0)	0.1
Performance status, n (%)			
0	890 (94.0)	71 (64.0)	<0.001
1	57 (6.0)	40 (36.0)	
Chronic liver diseases, n (%)			
HCV	384 (40.6)^a^	51 (45.9)	0.06
HBV	147 (15.5)	10 (9.0)	
HCV plus HBV	2 (0.2)	0 (0.0)	
Alcohol	97 (10.3)	19 (17.1)	
Others	316 (33.4)	31 (27.9)	
Viral-related disease, n (%)	533 (56.3)	61 (55.0)	0.8
Child-Pugh class, n (%)			
A	899 (94.9)	102 (91.9)	0.2
B	48 (5.1)	9 (8.1)	
ALBI score			
Median (IQR)	−2.78 [−3.04, −2.50]	−2.60 [−2.82, −2.25]	<0.001
mALBI grade, n (%)			
1	621 (65.6)	56 (50.5)	0.001
2a	194 (20.5)	25 (22.5)	
2b	123 (13.0)	30 (27.0)	
3	9 (1.0)	0 (0.0)	
Solitary nodule, n (%)	729 (77.0)	104 (93.7)	<0.001
Maximum tumor size (cm)			
Median (IQR)	3.3 [2.2, 5.3]	3.7 [2.6, 5.6]	0.07
≥4 cm	369 (39.0)	54 (48.6)	0.05
MVI, n (%)			
Presence	80 (8.4)	5 (4.5)	0.2
AFP			
≥100 ng/mL	206 (22.2)^b^	26 (23.6)	0.7
DCP			
≥100 mAU/mL	425 (46.7)^c^	46 (41.8)	0.4

^a^ Data were missing for 1 patient.

^b^ Data were missing for 20 patients.

^c^ Data were missing for 38 patients.

Abbreviations: AFP, α-fetoprotein; ALBI score, albumin-bilirubin score; CIRT, carbon-ion radiotherapy; DCP, des-gamma-carboxy prothrombin; mALBI grade, modified albumin-bilirubin grade; MVI, macroscopic vascular invasion; SR, surgical resection.

After IPTW weighting, there were no significant differences in patient characteristics between the 2 groups. The details of the IPTW cohort are summarized in Supplemental Table S1, http://links.lww.com/HC9/C106.

### Comparison of RFS and OS between the SR and CIRT groups

**FIGURE 2:**
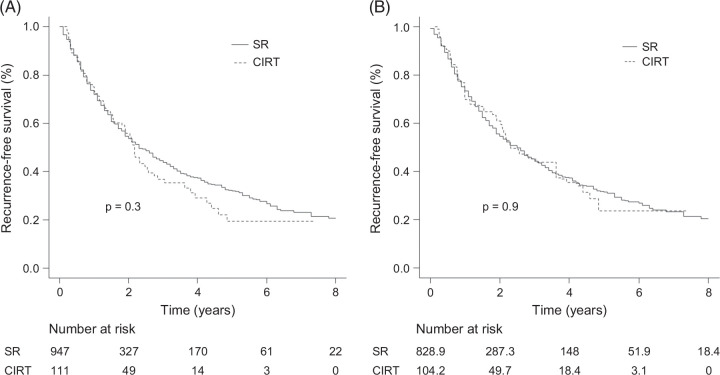
RFS in the SR and CIRT groups. (A) Before IPTW analysis, the median RFS was 2.3 years (95% CI: 2.1–2.7) in the SR group and 2.2 years (95% CI: 1.8–2.5) in the CIRT group, without statistical significance (*p*=0.3). (B) After IPTW analysis, the median RFS was 2.5 years (95% CI: 2.1–2.9) in the SR group and 2.3 years (95% CI: 1.6–4.3) in the CIRT group, which did not reach statistical significance (*p*=0.9). Abbreviations: CIRT, carbon-ion radiotherapy; IPTW, inverse probability of treatment weighting; RFS, recurrence-free survival; SR, surgical resection.

The median observation period was 3.3 years (IQR: 1.4–5.8) in the SR group and 2.8 years (IQR: 1.6–4.5) in the CIRT group, with no statistical significance (*p*=0.2). Before IPTW analysis, the median RFS was 2.3 years (95% CI: 2.1–2.7) in the SR group and 2.2 years (95% CI: 1.8–2.6) in the CIRT group, with no statistical significance (*p*=0.3). After IPTW analysis, the median RFS was 2.5 years (95% CI: 2.1–2.9) in the SR group and 2.3 years (95% CI: 1.6–4.3) in the CIRT group, which remained statistically nonsignificant (*p*=0.9; Figures 2A, B). To confirm the robustness of the IPTW results, we additionally performed PSM. In the matched cohort (n=267), the median RFS was 1.7 years (95% CI: 1.4–2.3) in the SR group (n=178) and 2.2 years (95% CI: 1.8–3.0) in the CIRT group (n=89), with no significant difference (*p*=0.3; Supplemental Figure 1A, http://links.lww.com/HC9/C107).

**FIGURE 3:**
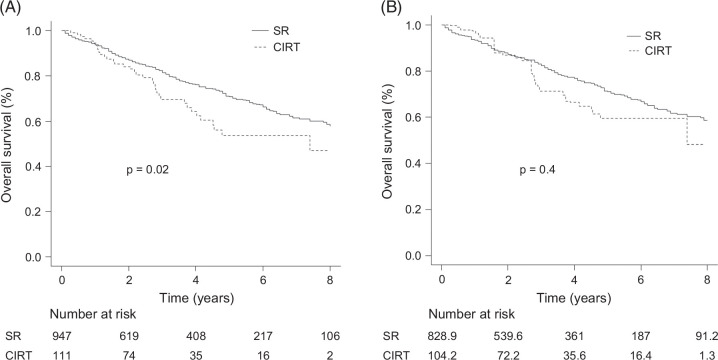
OS in the SR and CIRT groups. (A) Before IPTW analysis, the median OS was not reached in the SR group, while it was 7.4 years (95% CI: 4.1–NA) in the CIRT group. The SR group showed better survival compared to the CIRT group (*p*=0.02). (B) After IPTW analysis, the median OS was not reached in the SR groups, whereas it was 7.4 years (95% CI: 3.7–NA) in the CIRT group. There was no significant difference in OS between the two groups (*p*=0.4). Abbreviations: CIRT, carbon-ion radiotherapy; IPTW, inverse probability of treatment weighting; NA, not applicable; OS, overall survival; SR, surgical resection.

Regarding OS analysis, the median OS was not reached in the SR group, with a 5-year survival rate of 70.7% (95% CI: 67.0–74.1), whereas it was 7.4 years (95% CI: 4.1–not applicable [NA]) in the CIRT group. The SR group demonstrated better survival compared to the CIRT group (*p*=0.02; Figure 3A). In the IPTW cohort, the median OS remained unreached in the SR group, with a 5-year survival rate of 71.1% (95% CI: 67.0–74.8), while it was 7.4 years (95% CI: 3.7–NA) in the CIRT group. No statistically significant difference in OS was observed between the 2 groups (*p*=0.4; Figure 3B). During the observation period, 250 patients (26.4%) in the SR group and 37 patients (33.3%) in the CIRT group died. The causes of death in the SR group were progression of HCC in 148 patients (59.2%), liver failure in 29 patients (11.6%), and other causes in 73 patients (29.2%). In the CIRT group, the corresponding numbers were 21 (56.8%), 2 (5.4%), and 14 (37.8%), respectively. The distribution of causes of death did not differ significantly between the 2 groups (*p*=0.4). In the PSM-matched cohort, the median OS was not reached in either group. The 5-year survival rate was 61.1% (95% CI: 52.0–69.0) in the SR group and 57.3% (95% CI: 43.3–69.1) in the CIRT group, with no significant difference between the groups (*p*=1.0; Supplemental Figure S1B, http://links.lww.com/HC9/C107).

In the crude cohort, univariate analyses identified several variables as potential predictors of RFS, including age, mALBI grade 2b or 3, multiple nodules, tumor diameter ≥4 cm, presence of MVI, serum AFP ≥100 ng/mL, and serum DCP ≥100 mAU/mL. Multivariate analysis confirmed that all of these factors were independently associated with shorter RFS: age (HR: 1.02, 95% CI: 1.01–1.03, *p*<0.001), mALBI grade 2b or 3 (HR: 1.37, 95% CI: 1.11–1.70, *p*=0.004), multiple nodules (HR: 1.31, 95% CI: 1.07–1.60, *p*=0.009), tumor diameter ≥4 cm (HR: 1.34, 95% CI: 1.11–1.61, *p*=0.002), presence of MVI (HR: 1.59, 95% CI: 1.19–2.12, *p*=0.002), serum AFP ≥100 ng/mL (HR: 1.26, 95% CI: 1.03–1.54, *p*=0.03), and serum DCP ≥100 mAU/mL (HR: 1.49, 95% CI: 1.23–1.80, *p*<0.001). However, treatment modality (CIRT) was not identified as an independent predictor of RFS (Table 2).

**TABLE 2:** Univariate and multivariate analyses associated with RFS in the crude cohort

	Univariate analysis		Multivariate analysis	
	Hazard ratio (95% CI)	*p*	Hazard ratio (95% CI)	*p*
Age				
Per years	1.02 (1.01–1.03)	<0.001	1.02 (1.01–1.03)	<0.001
Sex				
Female	1.07 (0.88–1.31)	0.5		
Performance status				
1	1.18 (0.90–1.54)	0.2		
Chronic liver diseases				
viral	1.08 (0.91–1.28)	0.4		
mALBI grade				
2b or 3	1.41 (1.14–1.74)	0.001	1.37 (1.11–1.70)	0.004
The number of tumors				
Multiple nodule	1.43 (1.18–1.73)	<0.001	1.31 (1.07–1.60)	0.009
Maximum tumor size				
≥4 cm	1.69 (1.42–1.99)	<0.001	1.34 (1.11–1.61)	0.002
MVI				
Presence	1.76 (1.39–2.32)	<0.001	1.59 (1.19–2.12)	0.002
AFP				
≥100 ng/ml	1.47 (1.21–1.78)	<0.001	1.26 (1.03–1.54)	0.03
DCP				
≥100 mAU/ml	1.84 (1.55–2.18)	<0.001	1.49 (1.23–1.80)	<0.001
Treatments				
CIRT	1.15 (0.89–1.48)	0.3		

Abbreviations: AFP, α-fetoprotein; CIRT, carbon-ion radiotherapy; DCP, des-gamma-carboxy prothrombin; mALBI grade, modified albumin-bilirubin grade; MVI, macroscopic vascular invasion; RFS, recurrence-free survival.

For OS, univariate analyses identified several variables as potential predictors, including age, PS, mALBI grade 2b or 3, multiple nodules, tumor diameter ≥4 cm, presence of MVI, serum AFP ≥100 ng/mL, serum DCP ≥100 mAU/mL, and treatment modality (CIRT). Multivariate analysis confirmed that age (HR: 1.03, 95% CI: 1.01–1.04, *p*<0.001), mALBI grade 2b or 3 (HR: 1.57, 95% CI: 1.16–2.12, *p*=0.004), multiple nodules (HR: 1.52, 95% CI: 1.15–2.01, *p*=0.003), tumor diameter ≥4 cm (HR: 1.53, 95% CI: 1.17–1.99, *p*=0.002), presence of MVI (HR: 2.05, 95% CI: 1.44–2.92, *p*<0.001), serum AFP ≥100 ng/mL (HR: 1.58, 95% CI: 1.21–2.08, *p*<0.001), and serum DCP ≥100 mAU/mL (HR: 1.85, 95% CI: 1.40–2.45, *p*<0.001) were independently associated with poorer OS. In contrast, PS and treatment modality (SR vs. CIRT) were not identified as independent predictors in the multivariate analysis (Table 3).

**TABLE 3:** Univariate and multivariate analyses associated with OS in the crude cohort

	Univariate analysis		Multivariate analysis	
	Hazard ratio (95% CI)	*p*	Hazard ratio (95% CI)	*p*
Age				
Per years	1.03 (1.02–1.05)	<0.001	1.03 (1.01–1.04)	<0.001
Sex				
Female	0.97 (0.74–1.30)	0.9		
Performance status				
1	1.72 (1.21–2.45)	0.003	1.19 (0.79–1.81)	0.4
Chronic liver diseases				
viral	1.12 (0.89–1.42)	0.3		
mALBI grade				
2b or 3	1.80 (1.36–2.38)	<0.001	1.57 (1.16–2.12)	0.004
The number of tumors				
Multiple nodule	1.68 (1.30–2.18)	<0.001	1.52 (1.15–2.01)	0.003
Maximum tumor size				
≥4 cm	2.22 (1.75–2.82)	<0.001	1.53 (1.17–1.99)	0.002
MVI				
Presence	2.63 (1.89–3.67)	<0.001	2.05 (1.44–2.92)	<0.001
AFP				
≥100 ng/ml	2.14 (1.65–2.77)	<0.001	1.58 (1.21–2.08)	<0.001
DCP				
≥100 mAU/ml	2.54 (1.98–3.26)	<0.001	1.85 (1.40–2.45)	<0.001
Treatments				
CIRT	1.50 (1.06–2.13)	0.02	1.35 (0.91–2.00)	0.1

Abbreviations: AFP, α-fetoprotein; CIRT, carbon-ion radiotherapy; DCP, des-gamma-carboxy prothrombin; mALBI grade, modified albumin-bilirubin grade; MVI, macroscopic vascular invasion; OS, overall survival.

### RFS and OS in patients with HCC with tumor diameter ≥4 cm in the SR and CIRT groups

Because CIRT for HCC with a tumor diameter ≥4 cm was approved for insurance reimbursement in Japan in 2022, we analyzed this patient subgroup separately. This subset included 369 patients in the CIRT group and 54 patients in the SR group.

**FIGURE 4:**
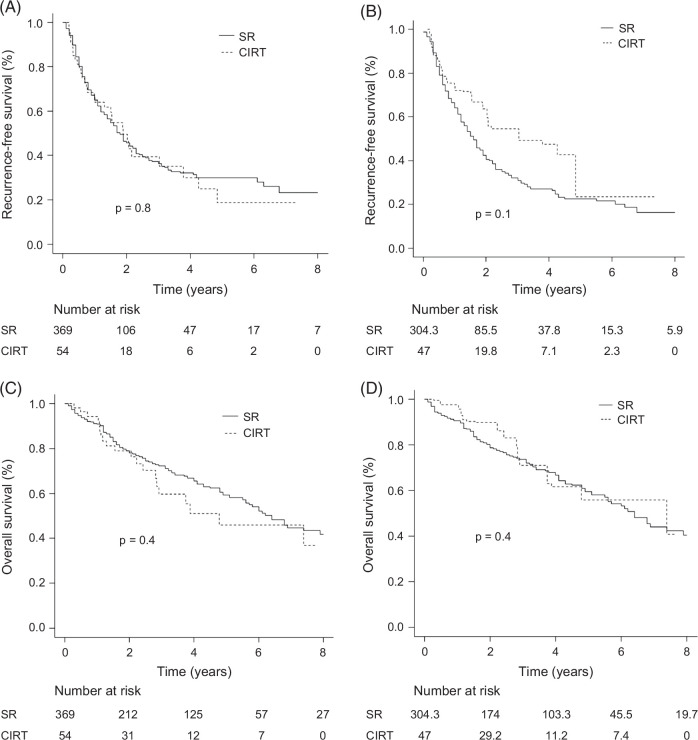
RFS and OS in patients with HCC with tumor diameter ≥4 cm in the SR and CIRT groups. (A) Before IPTW analysis, the median RFS was 1.5 years (95% CI: 1.3–1.9) in the SR group and 1.9 years (95% CI: 1.0–3.0) in the CIRT group, without statistical significance (*p*=0.8). (B) After IPTW analysis, the median RFS was 1.6 years (95% CI: 1.3–1.9) and 3.0 years (95% CI: 1.6–NA) years in the SR and CIRT groups, respectively, which did not reach statistical significance (*p*=0.1). (C) Before IPTW analysis, the median OS was 6.4 years (95% CI: 5.6–7.9) in the SR group and 4.8 years (95% CI: 2.8–NA) in the CIRT group, without statistical significance (*p*=0.4). (D) After IPTW analysis, the median OS was 6.4 years (95% CI: 5.5–7.9) in the SR group and 7.4 years (95% CI: 2.9–NA) in the CIRT group, which did not reach statistical significance (*p*=0.4). Abbreviations: CIRT, carbon-ion radiotherapy; IPTW, inverse probability of treatment weighting; NA, not applicable; OS, overall survival; RFS, recurrence-free survival; SR, surgical resection.

The median observation period was 2.5 years (IQR: 1.2–5.1) in the SR group and 2.1 years (IQR: 1.1–3.7) in the CIRT group, with no statistical significance (*p*=0.5). In the crude cohort, the median RFS was 1.5 years (95% CI: 1.3–1.9) in the SR group and 1.9 years (95% CI: 1.0–3.0) in the CIRT group, showing no statistical significance (*p*=0.8). In the IPTW cohort, the median RFS was 1.6 years (95% CI: 1.3–1.9) in the SR group and 3.0 years (95% CI: 1.6–NA) in the CIRT group, with no significant difference observed (*p*=0.1). The Kaplan-Meier curves are shown in Figures 4A, B.

Before IPTW analysis, the median OS was 6.4 years (95% CI: 5.6–7.9) in the SR group and 4.8 years (95% CI: 2.8–NA) in the CIRT group, with no significant difference (*p*=0.4; Figure 4C). After the IPTW adjustment, the median OS was 6.4 years (95% CI: 5.5–7.9) in the SR group and it was 7.4 years (95% CI: 2.9–NA) in the CIRT group, showing no significant difference (*p*=0.4; Figure 4D).

### Adverse events and impact on liver function in the CIRT group

Next, we focus on treatment-related adverse events and changes in liver function in the CIRT group. Grade ≥3 adverse events were observed in 7 patients (6.3%). Bile duct infection, hepatic encephalopathy, and gastrointestinal bleeding were observed in 3 (2.7%), 2 (1.8%), and 2 (1.8%) patients, respectively. Among the 61 patients with virus-related liver disease (51 with HCV and 10 with HBV), 18 (35.3%) of the HCV-positive patients had achieved sustained virological response (SVR), and 7 (70.0%) of the HBV-positive patients had received nucleos(t)ide analog therapy before CIRT. No cases of liver injury due to HCV flare or HBV reactivation were observed.

**FIGURE 5:**
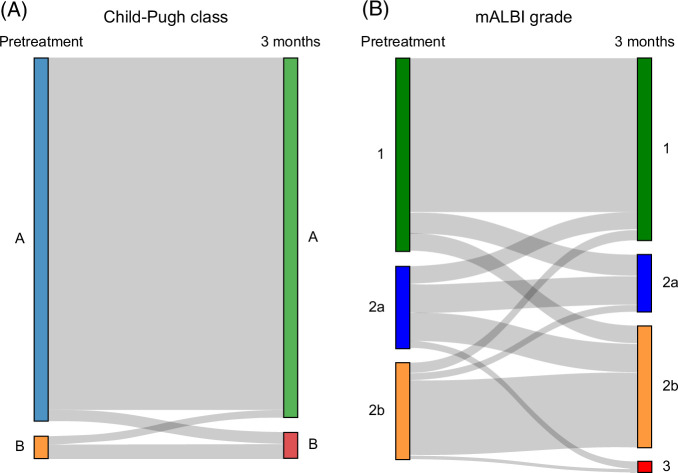
Changes in liver function at baseline and 3 months after CIRT. Liver function data at 3 months were missing for 8 patients; therefore, 103 patients were included in the analysis. (A) A Sankey diagram shows that among 97 patients classified as Child-Pugh class A at the initiation of CIRT, 3 (3.1%) progressed to class B, while the remaining 94 (96.9%) maintained class A liver function. Of the 6 patients with class B liver function at baseline, 2 (33.3%) improved to class A and 4 (66.7%) remained in class B. No patients progressed to Child-Pugh class C. (B) Regarding evaluation by mALBI grade, among 54 patients with mALBI grade 1 at baseline, 6 (11.1%) and 5 (9.3%) patients progressed to mALBI grades 2a and 2b, respectively, while the remaining 43 (79.6%) maintained mALBI grade 1 liver function. Of the 22 patients with mALBI grade 2a, 5 (22.7%) improved to grade 1, 7 (31.8%) maintained grade 2a, 8 (36.4%) progressed to grade 2b, and 2 (9.1%) progressed to grade 3. Among 27 patients with mALBI grade 2b, 3 (11.1%) and 2 (7.4%) improved to grades 1 and 2a, respectively, 21 (77.8%) maintained grade 2b, and 1 (3.7%) progressed to grade 3. Overall, 81 patients (78.6%) maintained or improved their liver function, while 22 patients (21.4%) experienced a deterioration in liver function, as assessed by mALBI grade. Abbreviations: CIRT, carbon-ion radiotherapy; mALBI grade, modified albumin-bilirubin grade.

We compared liver function between the pretreatment period and 3 months after CIRT. Liver function data at 3 months were missing for 8 patients; therefore, 103 patients were included in the analysis. Among 97 patients classified as Child-Pugh class A at the initiation of CIRT, 3 (3.1%) progressed to class B, while the remaining 94 (96.9%) maintained class A liver function. Of the 6 patients with class B liver function at baseline, 2 (33.3%) improved to class A and 4 (66.7%) remained in class B. No patients progressed to Child-Pugh class C (Figure 5A). Regarding evaluation by mALBI grade, among 54 patients with mALBI grade 1 at baseline, 6 (11.1%) and 5 (9.3%) patients progressed to mALBI grades 2a and 2b, respectively, while the remaining 43 (79.6%) maintained mALBI grade 1 liver function. Of the 22 patients with mALBI grade 2a, 5 (22.7%) improved to grade 1, 7 (31.8%) maintained grade 2A, 8 (36.4%) progressed to grade 2b, and 2 (9.1%) progressed to grade 3. Among 27 patients with mALBI grade 2b, 3 (11.1%) and 2 (7.4%) improved to grades 1 and 2a, respectively, 21 (77.8%) maintained grade 2b, and 1 (3.7%) progressed to grade 3. Overall, 81 patients (78.6%) maintained or improved their liver function, while 22 patients (21.4%) experienced a deterioration in liver function, as assessed by mALBI grade (Figure 5B).

## DISCUSSION

The major findings of the present study were that the patients in the CIRT group were significantly older, had worse PS, had worse liver function, and more frequently had solitary HCC compared to those in the SR group. To correct the imbalance between the 2 groups, we conducted the ITPW analysis, showing that the significant difference in the RFS was not observed between both groups in the crude cohort (*p*=0.3) and the IPTW cohort (*p*=0.9). While the patients in the CIRT groups showed worse survival compared to those in the SR group before IPTW analysis (*p*=0.02), the difference did not reach to statistically significant in the IPTW cohort (*p*=0.4). Multivariate analyses revealed that treatment modality (CIRT) was not identified as a predictive factor for both RFS or OS. Adverse events associated with CIRT were generally considered tolerable, with liver function preserved in many patients. Given these results, the CIRT treatment was comparable to the SR. To our knowledge, this is the first study demonstrating that the CIRT could be one of the curative treatments for patients with localized HCC.

To contextualize our findings, we reviewed previous studies evaluating the efficacy and safety of CIRT in patients with HCC. A pooled analysis of two prospective studies^7^ demonstrated local control rates of 94.7%, 91.4%, and 90.0% at 1, 3, and 5 years, respectively. Importantly, no treatment-related serious adverse events were reported in this analysis.^7^ A prospective observational study analyzing 4 fractions of CIRT for HCC reported that the local control rates were 92.6%, 76.5%, and 76.5% at 2, 3, and 4 years, respectively, with the median progression-free survival of 25.6 months.^9^ Grade 3 acute or late adverse events were reported in 2 cases.^9^ These findings suggest that CIRT offers excellent local tumor control with a favorable safety profile, supporting its potential as an effective treatment option for HCC. However, it should be noted that these studies were a single-arm design, and comparative data evaluating the outcomes of CIRT against other treatment modalities remain limited.

Regarding previous studies comparing outcomes of charged particle therapy and standard treatment for HCC, a retrospective study reported no significant differences in RFS and OS between proton beam therapy (PBT) and SR after propensity score matching.^12^ Another retrospective study conducted in Japan revealed that RFS and OS in patients receiving PBT were comparable to those in patients receiving RFA.^13^ A phase 3 randomized controlled trial^14^ comparing PBT and RFA showed no significant differences in local control, RFS and OS, demonstrating that PBT showed almost the same clinical benefit compared to RFA. With respect to CIRT, a retrospective study^15^ reported that the RFS and OS of patients treated with CIRT were comparable to those of RFA. However, there are a few reports comparing the efficacy of CIRT and SR. Further studies comparing CIRT with standard treatments are warranted to clarify its clinical efficacy.

The results of the NRG/RTOG 1112 trial have recently been reported.^16^ This phase 3 randomized controlled trial evaluated the efficacy of sorafenib plus SBRT compared to sorafenib alone in patients with unresectable HCC.^16^ Although the addition of SBRT showed a clinically meaningful improvement over sorafenib alone, the difference did not reach statistical significance.^16^ Notably, approximately 70% of patients in this trial had MVI, suggesting that SBRT may be particularly beneficial in this subgroup. Given that particle therapy may reduce the incidence of nonclassic radiation-induced liver damage compared to SBRT due to the Bragg peak phenomenon and the absence of a low-dose bath distal to the target beam path, the combination of CIRT and systemic therapy may be beneficial for patients with unresectable HCC and MVI. Results from the currently ongoing randomized controlled trial (NRG-GI003), which compares the efficacy of PBT and SBRT, are eagerly awaited. In the present study, approximately 8% of patients had MVI. A previous study^17^ reported that patients with MVI who received CIRT achieved better local tumor control but experienced a high rate of distant metastasis after treatment. Based on the results of the IMbrave050 trial,^18^ adjuvant systemic therapy following CIRT may be beneficial for patients with MVI. Further studies are warranted to determine the optimal treatment strategies, including radiation therapy, for patients with MVI.

The present study had several limitations. First, it was conducted retrospectively. Second, the sample size was relatively small, particularly the number of patients receiving CIRT, necessitating future studies with larger cohorts. Third, there were imbalances in patient characteristics between the 2 groups. Although IPTW and PSM analysis was performed to adjust for confounding factors, unmeasured variables such as comorbidities and anatomical tumor location could not be accounted for. Considering that CIRT is usually performed for unresectable HCC, these unmeasured variables might have contributed to the poorer clinical outcomes observed in the CIRT group.

In conclusion, CIRT showed no statistically significant differences in RFS or OS compared to SR, suggesting its potential as a curative treatment option for early-stage HCC.

## Supplementary Material

**Figure:**